# Comparison of fracture strength in implant supported zirconia-titanium base abutments

**DOI:** 10.6026/973206300200665

**Published:** 2024-06-30

**Authors:** Rupa Singh, Trishala Ajit Patel, Asish Kumar Barui, Manu Verma, Jenifer Kerketta, Prasanta Majumder

**Affiliations:** 1 Department of Prosthodontics and Crown & Bridge, Mithila Minority Dental College and Hospital, Darbhanga, Bihar, India; 2 Prosthodontics and Crown & Bridgework and Oral Implantology, Faculty of dental science, Dharmsinh Desai University, Nadiad, Gujarat, India; 3 MDS,MAS, Prosthodontist, Metro Dental Spa ,Kolkata, India; 4 MDS in Oral and Maxillofacial Prosthodontics and Implantology, Government Dental College, Raipur, Chhattisgarh, India; 5 Department of Public Health Dentistry, Agartala Government Dental College and IGM Hospital, Agartala, Tripura, India

**Keywords:** Implant, abutment, polishing, modification, fracture resistance

## Abstract

The fracture strength of implant supported zirconia-titanium base (Zi-Ti) base restorations with and without modification of submucosal cervical contour is of interest to dentists. 80 zirconia specimens were adjusted onto the Ti-base. One category consisted of specimens that underwent modification. Other category consisted of abutments without modification. There was polishing and recon touring at the interface of Zi-Ti base in cervical regions. Using the universal testing apparatus fracture resistance was assessed for every sample in every category in Newtons (N). The fracture strength of abutments with modification ranged between 4465.79 - 6523.50 N with mean value of 5604.24 ± 497.62 N. On the other hand, values of fracture strength varied between 5511.42 - 7064.33 N. in abutments without modification with mean fracture strength values of 6265.95 ± 331.61. It was observed that the fracture strength was lesser in abutments that underwent modification.

## Background:

Among the most significant variables in establishing the ideal soft as well as hard tissues is the emergence profile [1, 2-3]. Specifically, in the esthetic region, dental implant restorations should have an emergence profile that closely resembles the natural teeth. Inadvertent contoured abutments can lead to undermined accessibility for oral hygiene and exacerbated soft tissue, which can produce anaesthetic results [4, 5-6]. Thus, it is crucial to create an appropriate shaped implant restoration with an acceptable emergence appearance as well as gingival architecture that complements the neighbouring teeth for both aesthetic and operational implant therapy [7, 8, 9, 10-11].The efficiency of titanium dental implants for replacing teeth in the oral cavity is well documented, as these abutments are biocompatible and have acceptable mechanical properties [12, 13- 14]. However, even when placed subgingivally, a dull grey background may compromise the esthetic results of these abutments. High implant survival rate and success rates in treating single, partial or total edentulous, the esthetic outcome has become the main focus of interest in aesthetically sensitive areas [15, 16-17]. Hence, all ceramic abutments were introduced in 1991 to evade discoloration at the cervical margin. Although these abutments show esthetical optimal results, their strength and fatigue resistance compared to metal abutments, remain a concern [18, 19, 20-21]. The benefits of aluminium oxide abutments include acceptable optical translucency, adequate shade, and proper alignment within the dental implant. They occasionally lack the strength to withstand the masticatory forces, unfortunately [22, 23- 24]. In addition, zirconia implant abutments are becoming more and more well-liked due to their color and degree of light transmittance, as well as their reputedly strong resistance to fracture [11- 14]. Zirconia abutments come in a variety of styles for use in dentistry. They might be customized or prefabricated. Prefabricated zirconia abutments are typically created utilizing computer aided design/computer assisted manufacturing (CAD/CAM) technologies [10-13]. These abutments may be built entirely or partially of zirconia. Prefabricated abutments can sometimes fail to give the ideal morphology and aesthetic, such as the required tooth size and soft tissue shapes, while being homogeneous, uniform, simple to use, and well-fitting [8- 12]. Implants placed subcrestally may cause problems with interproximal bone closeness that affect the submucosal morphology of zirconia restorations supported by implants when the restorations are being delivered [4- 7]. The mesial distal submucosal portions may need to be modified in order to adequately seat the restoration while avoiding the interproximal bone to rub against it. There are zirconia abutments for clinical use, including those made by copy-milling process, although there aren't many lab researches examining these abutment assemblies' resistance to fractures [5-8]. Moreover, the influence of modification of submucosal cervical contour of implant supported zirconia-titanium base (Zi-Ti base) restorations in fracture strength is not explored [11-16]. Therefore, this in vitro study was carried out to compare fracture strength of implant supported Zi-Ti base restorations with and without modification of submucosal cervical contour.

## Materials and Methods:

Implant Zi-Ti abutments created in the shape of a maxillary premolar were prepared for the Straumann implant lab analog. Zirconia specimens were adjusted onto the Ti-base. 80 specimens were prepared. They were divided into two categories. One category consisted of specimens that underwent modification. Other category consisted of abutments without modification. Each category consisted of 40 specimens (Table 1).There was polishing and recontouring at the interface of Zi-Ti base in cervical regions. The abutments were then submerged in the colored cove ring liquid and dried below a red lamp. After that, the zirconia abutments underwent an 8-hour sintering treatment in a sintering oven approximately at 1,500°C. As directed by the manufacturer, a 24 N/cm torque was used to attach each abutment to its matching implant. The term "abutment assembly" was subsequently used when referring to the abutment, abutment screw and implant conjunction. To hold the specimens in place while the implant fixture's long axis was tilted 30 degrees, a stainless steel jig was made. A small layer (0.1 mm) of Mylar film was placed between the loading stylus and the zirconia abutment to further control loading and minimizes unintentional surface damage. The occlusal surface was subjected to a vertical stress (crosshead speed = 0.1 mm/min). At this speed, the load rose until failure happened. Using the universal testing apparatus (Germany 2050, Zuick/Roell), fracture resistance was assessed for every sample in every category in Newtons (N).

## Statistical analysis:

Kolmogorov-Smirnov test was used to check for a normal distribution of the data. The impact of polishing and recontouring on the specimens' fracture resistance was evaluated using univariate and post-hoc analyses. A 5% threshold for statistical significance was established. Applying a 2-tailed t test for separate specimens, we evaluated differences between modified implant restorations and unmodified implants restorations.

## Results:

The fracture strength of abutments with modification ranged between 4465.79 - 6523.50N with mean value of 5604.24 ± 497.62N. On the other hand, values of fracture strength varied between 5511.42 - 7064.33 N in abutments without modification with mean fracture strength values of 6265.95 ± 331.61N. It was observed that the fracture strength was lesser in abutments that underwent modification. The findings were statistically significant (p=0.001) (Table 2).

## Discussion:

The residual alveolar bone, the soft tissue surrounding the implant, and the crown form are the three factors that are utilized to characterize the appearance and general wellness of implant restorations [5-12]. Both function and aesthetics require consideration of these elements. The physiological crown contour is one of these elements that, in terms of prosthesis, is crucial for preserving the periodontal health surrounding an implant by encouraging the self-cleaning activity [2-6]. A bulky prosthesis or a crown breakage that alters the crown's shape will disrupt the original shape of the crown and impair easy chewing. Food lodgement, plaque build-up surrounding the implant, and possible periodontal issues will result from it. An implant abutment restoration will also experience this effect [4-8]. The emergence profile is one of the most important factors in determining the optimal soft and hard tissues. Dental implant restorations should, in particular, emerge with a profile that closely mimics that of natural teeth in the esthetic region [12, 19]. Unintentionally shaped abutments can worsen soft tissue and reduce accessibility for dental hygiene, both of which can have an unattractive effect [11-14]. For both cosmetic and functional implant therapy, it is therefore essential to design an implant restoration that is suitably formed, has a respectable emergence appearance, and has gingival architecture that blends in with the surrounding teeth [19- 23].

This in vitro study was carried out to compare fracture strength of implant supported zirconia-titanium base (Zi-Ti base) restorations with and without modification of submucosal cervical contour. It was observed that the fracture strength was lesser in abutments that underwent modification. The fracture strength of abutments with modification ranged between 4465.79 - 6523.50 N with mean value of 5604.24 ± 497.62 N. On the other hand, values of fracture strength varied between 5511.42 - 7064.33N in abutments without modification with mean fracture strength values of 6265.95 ± 331.61 N. The findings were significant statistically (p=0.001). There are some studies which also support findings of our study because they have also shown that recontouring and polishing affect the fracture resistance of implant abutments[16-24]. However, some studies don't find significant effect on fracture resistance because these studies stated that fracture resistance after modification of implant abutment was sufficient to counter the masticatory process[21, 25-26].There are numerous designs of zirconia abutments available for use in dentistry. They could be prefabricated or customized. The most common method used to make prefabricated zirconia abutments is computer assisted design/computer assisted manufacturing (CAD/CAM) [11- 19]. Zirconia may be used wholly or in part to construct these abutments. Even while prefabricated abutments are homogeneous, uniform, easy to use, and well-fitting, they may not always provide the ideal morphology and aesthetic, such as the necessary tooth size and soft tissue forms [20-24]. Although there are zirconia abutments for clinical use, including those produced using copy-milling, there aren't many lab studies that look at the fracture resistance of these abutment assemblies [19-24].

According to a study, there was no discernible decrease in the simulated implant assemblies' resistance to fracture when zirconia abutments were prepared. It appears that the body boundaries are not as crucial as the connecting area's dimension [13- 18]. All implant abutments cracked in their investigation at rates greater than the maximal incisal forces thought to happen in the oral cavity anterior region. The results of the current study for Zi-Ti abutments, which show an acceptable clinical performance, are consistent with those of the study for conventional abutments [12- 16]. The implant-abutment assembly may fail in one or more places. The fact that these fractures appear at particular sites suggests that there may be a concentration of stress due to geometrical (design) factors [8- 14].

Because titanium dental implants' abutments are biocompatible and have respectable mechanical qualities, their effectiveness in replacing teeth in the oral cavity has been extensively studied [6-11]. Nevertheless, a dull gray backdrop could detract from the aesthetic appeal of these abutments even when positioned subgingivally. Because single, partial, or total edentulism can be successfully treated with excellent implant survival and success rates, the main emphasis of attention in esthetically sensitive areas has shifted to the aesthetic result [6- 9]. In order to prevent discolouration at the cervical margin, all-ceramic abutments were introduced in 1991. While these abutments exhibit ideal aesthetic results, questions still exist regarding their strength and fatigue resistance in comparison to metal abutments [10-15].

Aluminum oxide abutments provide the advantages of sufficient shade, appropriate optical translucency, and correct alignment inside the dental implant. Unfortunately, there are times when they are not strong enough to resist the masticatory forces [3- 8]. Zirconia implant abutments are also become more and more popular because of their color and level of light transparency in addition to their supposed great fracture resistance [6- 11]. This study confirms that the failure mechanisms identified were unique to both the construction and the material of the abutment. According to a study, the abutment/analog interface appeared to be the weakest part of the abutment assemblies [12-19]. According to certain research, zirconia abutment assemblages are most prone to fail at the cervical part of the abutment. Because of the levering effects, it is hypothesized that this location has the greatest amount of stress and torque [14- 17]. According to a study, the zirconia abutments failed due to fracture in the apical area of the abutment. This is also in line with findings from other research projects [18- 26]. When zirconia restorations are being delivered, subcrestally positioned implants may result in issues with interproximal bone proximity that impact the submucosal morphology of the restorations [14- 17]. To properly seat the restoration and keep the interproximal bone from rubbing against it, the mesial distal submucosal sections would need to be polished and recontoured [18- 23].

## Conclusion:

The fracture strength was lesser in implant abutments that were polished and modified as compared to implant abutment that was not modified.

## Figures and Tables

**Table 1:** Distribution of study specimens

**Category**	**Specimens**	**Number**
Category 1	Zi-Ti implant abutments with modification	40
Category 2	Zi-Ti implant abutments without modification	40

**Table 2:** Comparison of fracture strength Zi-Ti abutments with modification and without modification

	**Fracture Strength (N)**	
	**Range**	**Mean± SD**
Zi-Ti abutments with modification	4465.79- 6523.50	5604.24 ± 497.62
Zi-Ti abutments without modification	5511.42 - 7064.33	6265.95 ± 331.61
F value	22.341	
P value	0.001	
